# A boreal invasion in response to climate change? Range shifts and community effects in the borderland between forest and tundra

**DOI:** 10.1007/s13280-014-0606-8

**Published:** 2015-01-09

**Authors:** Bodil Elmhagen, Jonas Kindberg, Peter Hellström, Anders Angerbjörn

**Affiliations:** 1 Department of Zoology, Stockholm University, 106 91 Stockholm, Sweden; 2 Department of Wildlife, Fish, and Environmental Studies, Swedish University of Agricultural Sciences, 901 83 Umeå, Sweden; 3 Swedish Association for Hunting and Wildlife Management, Öster-Malma, 611 91 Nyköping, Sweden

**Keywords:** Climate change, Land-use change, Range shifts, Population cycles, Mammalia, Aves

## Abstract

It has been hypothesized that climate warming will allow southern species to advance north and invade northern ecosystems. We review the changes in the Swedish mammal and bird community in boreal forest and alpine tundra since the nineteenth century, as well as suggested drivers of change. Observed changes include (1) range expansion and increased abundance in southern birds, ungulates, and carnivores; (2) range contraction and decline in northern birds and carnivores; and (3) abundance decline or periodically disrupted dynamics in cyclic populations of small and medium-sized mammals and birds. The first warm spell, 1930–1960, stands out as a period of substantial faunal change. However, in addition to climate warming, suggested drivers of change include land use and other anthropogenic factors. We hypothesize all these drivers interacted, primarily favoring southern generalists. Future research should aim to distinguish between effects of climate and land-use change in boreal and tundra ecosystems.

**Electronic supplementary material:**

The online version of this article (doi:10.1007/s13280-014-0606-8) contains supplementary material, which is available to authorized users.

## Introduction

The global mean temperature has increased by 0.89 °C since 1901 (IPCC 2013). The Arctic experienced an above-average rate of warming during this period, and observed ecosystem change includes a prolonged growing season, changes in species phenology, northern advancement of southern species, and retreat of Arctic species (Post et al. 2009). These changes could be expected as a warmer climate should increase primary productivity in temperature-limited ecosystems. This could constrain species adapted to present temperature and resource conditions, while it could allow species limited by those conditions to increase in abundance and expand their distribution. In addition to such bottom-up effects, Callaghan et al. (2004) suggested that Arctic species are adapted to cope with physically harsh conditions at the expense of competitive ability, which would make them prone to decline in response to interactions with invading boreal species. For example, Hersteinsson and Macdonald (1992) suggested that an Arctic specialist, the arctic fox (*Vulpes lagopus*), should retreat in response to climate warming as its southern distribution limit should be determined by competition, while the northern distribution limit of its larger competitor, the red fox (*Vulpes vulpes*), should be determined by harsh climate conditions and low resource availability.

**Fig. 1:**
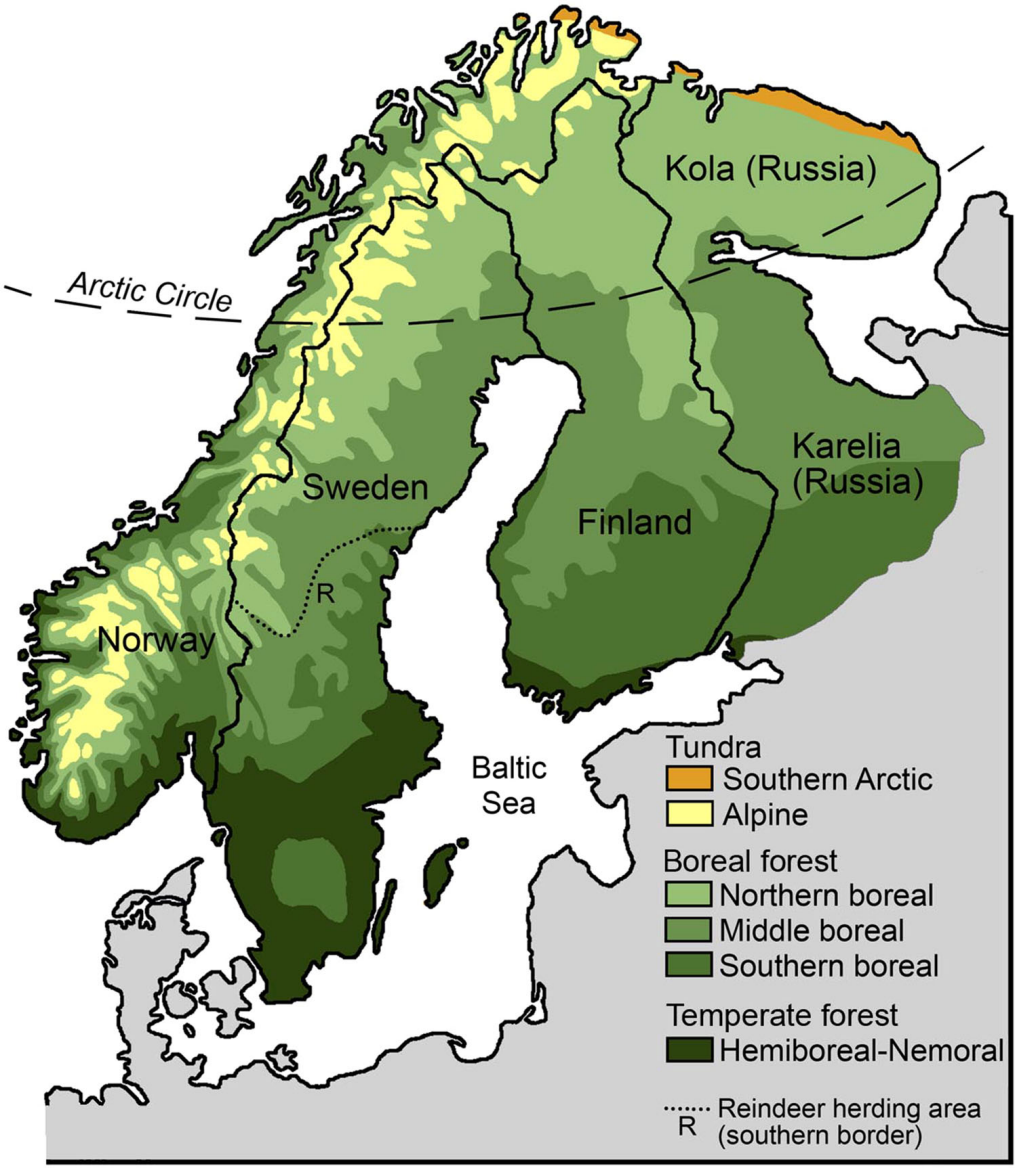
Vegetation zones in Fennoscandia. After: Moen (1999) and Ahti et al. (1968)

**Fig. 2:**
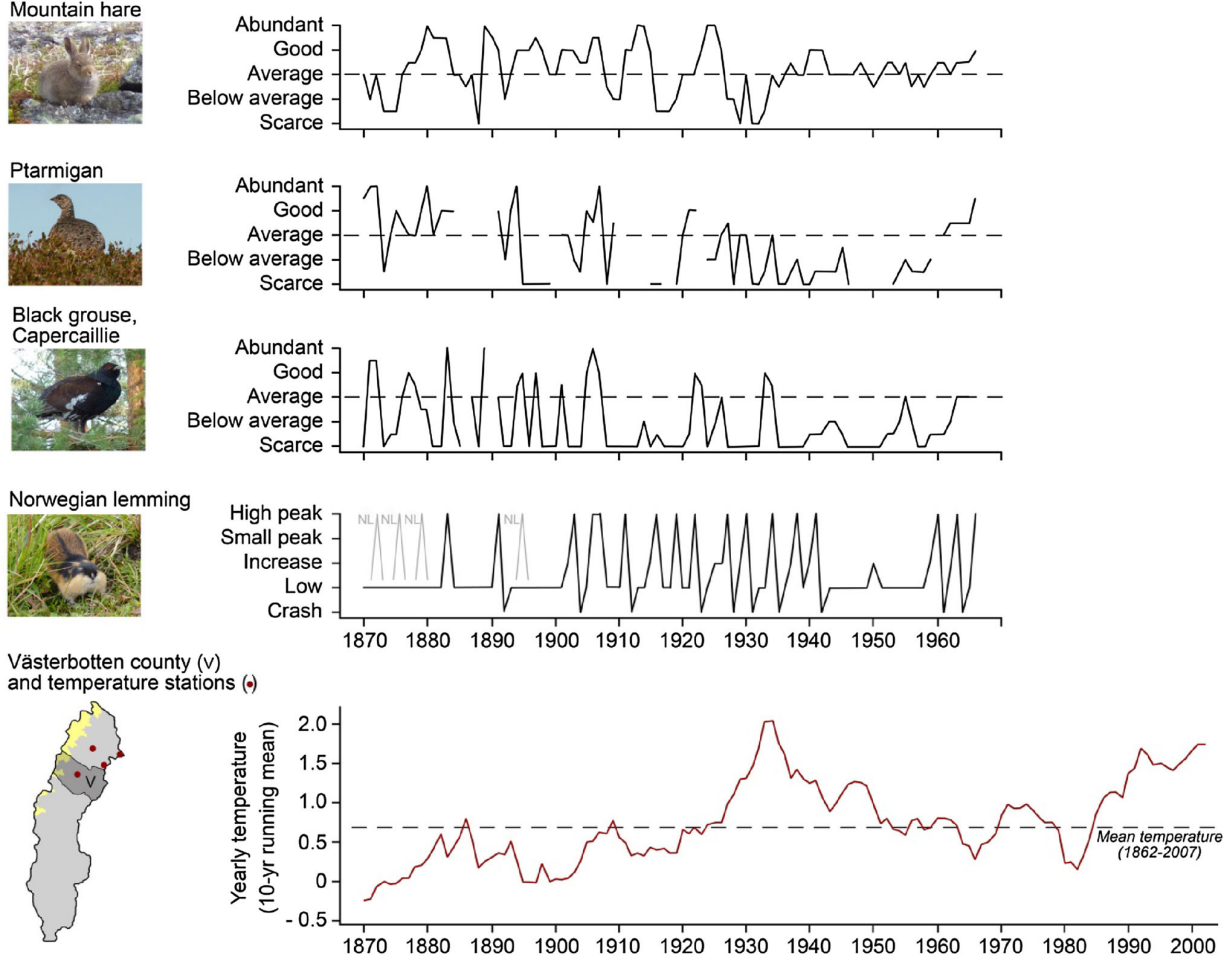
Yearly temperature (1870–2002) and long-term dynamics in mountain hare, rock ptarmigan/willow ptarmigan, black grouse/capercaillie, and Norwegian lemming (1870–1966) in Västerbotten county (V). We derived yearly status of hare and grouse from qualitative descriptions in Sweden’s Official Statistics (1870–1966) for Västerbotten county or the closest reported area (gaps = no information). Yearly lemming status in Västerbotten is taken from Angerbjörn et al. (2001), who assessed qualitative information from different regions in Scandinavia. The absence of high-abundance peaks in Västerbotten 1940–1960 was well supported, while absent peaks prior to 1900 could be related to information deficiency. However, peaks may have occurred in Västerbotten prior to 1900 as some peaks are known to have occurred in adjacent Nordland (NL)

Although climate change is projected to become the primary driver of biodiversity change in Arctic and boreal ecosystems in the next century, land-use change is projected to be almost as important in the boreal biome (Sala et al. 2000). This suggests that both drivers must be taken into account to understand change in biodiversity and ecosystem functioning. The Scandinavian mountain range runs south from the Arctic tundra, providing tundra conditions at high altitudes, but a high level of fragmentation by forested valleys creates a substantial interface between alpine tundra and boreal forest (Fig. 1). The mean temperature in Scandinavia has increased significantly since 1901, by 0.75–1.5 °C (IPCC 2013), with particularly warm periods in 1930–1950 and after 1980 (Fig. 2). However, human land use also intensified, in particular in boreal Sweden. The indigenous Sami developed an economy based on hunter/gathering, fur trade, and small-scale reindeer (*Rangifer tarandus*) husbandry at least one millennium ago. Reindeer-keeping practices transitioned into large-scale nomadic reindeer pastoralism in the early seventeenth century, and to extensive reindeer herding for meat production in the late nineteenth century (Lundmark 2007). In Sweden, the Sami economy was the primary land use in the north-west interior until the eighteenth century. At that time, the rate of agricultural expansion from the south and east coast increased, and agriculture reached the boreal–alpine interface in the nineteenth century (Anonymous 2006). Large-scale forestry based on selective logging of large trees developed in the mid-nineteenth century, followed by clear-cutting practices that have dominated forestry since the 1950s (Axelsson 2001). Hence, over the last 200 years, both climate and land use changed in northern Sweden.

Here, we review observed changes in the bird and mammal community in boreal and alpine Sweden since the nineteenth century, as well as suggested drivers of change. If climate warming has been an important driver of change, we expect southern species to have advanced their distribution and/or increased in abundance more often than northern species which rather should have retreated or declined. However, by also reviewing suggested drivers of change, we aim to explore whether additional drivers may have been important.

## Materials and methods

We focused primarily on species that have a distribution limit in the boreal or alpine biome, as range shifts can be observed only in these species. Furthermore, because we reviewed observed changes since the nineteenth century, some information sources were qualitative and/or anecdotal. We assumed that such sources were more likely to report substantial changes such as range shifts, although we also included information on population trends. We defined species as ‘southern’ or ‘northern’ depending on their distribution relative to their distribution limit. Southern species had a northern and/or high altitudinal distribution limit in the boreal forest or alpine tundra in Fennoscandia (Fig. 1), while northern species had a southern and/or low altitudinal distribution limit in boreal or alpine Sweden. Regarding the northern distribution limit, we used the Fennoscandian distribution limit to not exclude southern species that presently have a distribution limit slightly north of Sweden, as these might have advanced north within Sweden since the nineteenth century.

We did a two-part review. The first part was an overview of known changes in southern and northern bird species. Of 196 species regularly breeding in boreal or alpine Sweden, 118 (60 %) were classified as either southern or northern (Table S1 in Electronic Supplementary Material). The remaining species had, for example, eastern or scattered distributions (Table S2 in Electronic Supplementary Material). Svensson et al. (1999) compiled information on long-term trends in Swedish bird species since the nineteenth century. For each southern or northern bird species, we used this source to find out whether the distribution limit had changed, or if not, whether there was another long-term population trend (Electronic Supplementary Material). Differences between southern and northern species were tested with Fisher’s exact test. For species that had shown range shifts or a long-term population trend, we also noted suggested drivers of change.

In the second part of the review, we focused on northern and southern mammals, as well as bird species that interact strongly with these mammals. From a functional perspective, these bird species belong to the same wildlife community as the mammals, and we present the results with all species grouped according to their primary trophic function (herbivore/prey or predator) as well as body size (small, medium-sized, large). We excluded mammalian insectivores because they are not known to substantially interact with the community delineated above, and due to information deficiency regarding long-term change in the main taxa, shrews (*Soricidae*), and bats (*Chiroptera*).

## Results

### Changes in distributions and population trends in southern and northern bird species since the nineteenth century

Range shifts or long-term population trends had been reported for 68 of the 118 species (Table 1). Range contraction was significantly more frequent in northern than southern species (Table 1; 15 and 0 %, respectively; *p* = 0.0014, *n* = 118). In contrast, range expansion were significantly less frequent in northern than southern species (Table 1; 3 and 33 %, respectively; *p* < 0.001, *n* = 118). Overall, range contraction or decline was reported for 48 % of the northern species and 20 % of the southern species (Table 1; *p* = 0.0030, *n* = 118), while range expansion or increase was reported for 3 % of the northern species and 40 % of the southern species (Table 1; *p* < 0.001, *n* = 118). Land use was suggested as a driver of change for 45 of the 68 species which either changed their distribution limit or increased/declined in abundance (66 %), while climate sensitivity was mentioned for three species (4 %; Table 1). Range expansions, primarily northern or north-western advance in southern species, were often suggested to be linked to historic expansions of agriculture, built-up areas, and forestry. Range contractions and declines were also often linked to land-use change, in particular shifts between traditional and present-day forms of land use which decreased the amount of open and wet habitats due to less mowing and livestock grazing, as well as forestry and drainage. Northern species that declined due to land-use change were only affected in the southern part of their range (Table 1).

**Table 1:** Long-term trends in northern (N) and southern (S) bird species breeding in alpine and boreal Sweden. Trends were assessed from Svensson et al. (1999) and are presented as the percentage of species which contracted or expanded their range. Alternatively, if no range shift was reported, the long-term trend was classified as declining, increasing, or ‘no long-term trend.’ The latter refers to species that showed stable dynamics, fluctuating dynamics without a directional long-term trend, locally different trends, or where there was no information available. For each species, drivers of change suggested by Svensson et al. (1999) are indicated by letters; land-use change due to agriculture (*L*_A_), drainage of wet habitats (*L*_D_), eutrophication (*L*_E_), forestry (*L*_F_), or other human-related factors, e.g., effects associated with built-up areas or feeding (*L*_O_), as well as change due to hunting (H), change in overwintering areas (W), predation (P), disrupted rodent dynamics (R), reindeer grazing (G), climate change (C), or unknown (U)

Change	% (and no.) of species		Species and suggested drivers of change (within parenthesis)
	N (*n* = 33)	S (*n* = 85)	
Range contraction	15 % (*n* = 5)	0 % (*n* = 0)	*Northern*: *Bubo scandiacus* (R), *Calidris falcinellus* (*L*_AD_), *Gallinago media* (H*L*_D_), *Lagopus lagopus* (U), *Turdus torquatus* (*L*_A_)
Decline	33 % (*n* = 11)	20 % (*n* = 17)	*Northern*: *Anser erythropus* (HPW), *A. fabalis* ^a^ (HD*L*_A_), *Aythya marila* (U), *Calidris temminckii* ^a^ (*L*_A_), *Charadrius morinellus* (HW), *Eremophila alpestris* (U), *Falco columbarius* ^a^ (*L*_A_), *Gavia stellata* ^a^ (*L*_AD_), *Lagopus muta* (G), *Pluvialis apricaria* ^a^ (*L*_AF_), *Tringa glareola* ^a^ (*L*_D_) *Southern*: *Anas crecca* (*L*_D_), *Anthus trivialis* (*L*_A_W), *Caprimulgus europaeus* (*L*_A_W), *Carduelis cannabina* (*L*_A_), *Cuculus canorus* (U), *Dendrocopos minor* (*L*_F_), *Hirundo rustica* (*L*_A_), *Jynx torquilla* (*L*_A_), *Lanius collurio* (*L*_A_W), *Mergus serrator* (*L*_E_), *Parus montanus* (*L*_F_), *Passer domesticus* (*L*_A_), *Pernis apivorus* (W), *Picus viridis* (*L*_AF_), *Phoenicurus phoenicurus* (*L*_F_), *Lyrurus* *tetrix* (*L*_D_), *T. urogallus* (*L*_DF_P)
Range expansion	3 % (*n* = 1)	33 % (*n* = 28)	*Northern*: *Numenius phaeopus* (U) *Southern*: *Acrocephalus scirpaceus* (*L*_AE_), *Carduelis chloris* (*L*_U_), *Charadrius dubius* (*L*_O_), *Chroicocephalus ridibundus* (U), *Corvus monedula* (*L*_AO_), *Cyanistes caeruleus* (*L*_AO_), *Cygnus olor* (*L*_E_C), *Fulica atra* (*L*_DE_), *Garrulus glandarius* (U), *Larus canus* (U), *Lophophanes cristatus* (C), *Numenius arquata* (*L*_AD_), *Parus major* U), *Periparus ater* (*L*_F_), *Phylloscopus sibilatrix* (U), *Pica pica* (*L*_AO_), *Podiceps cristatus* (*L*_E_), *Poecile montanus* (*L*_O_), *Prunella modularis* (*L*_F_), *Pyrrhula pyrrhula* (U), *Sitta europaea* (U), *Sturnus vulgaris* (*L*_A_), *Sylvia atricapilla* (*L*_A_), *S. borin* (U), *S. curruca* (*L*_F_), *Tringa ochropus* (U), *Turdus merula* (U), *Vanellus vanellus* (U)
Increase	0 % (*n* = 0)	7 % (*n* = 6)	*Southern*: *Anas platyrhynchos* (U), *Apus apus* (*L*_O_), *Columba palumbus* (*L*_AO_), *Dendrocopos major* (*L*_FO_), *Motacilla flava thunbergi* (northern subspecies) (*L*_F_), *Troglodytes troglodytes* (*L*_F_C)
No long-term trend or no data	48 % (*n* = 16)	40 % (*n* = 34)	*Northern*: *Anas penelope*, *Anthus cervinus*, *Buteo lagopus*, *Calcarius lapponicus*, *Calidris alpina alpina* (northern subspecies), *C. maritima*, *Carduelis flammea flammea* (northern subspecies), *C. hornemanni*, *Clangula hyemalis*, *Falco rusticolus*, *Limosa lapponica*, *Luscinia svecica*, *Melanitta nigra*, *Phalaropus lobatus*, *Plectrophenax nivalis*, *Stercorarius longicaudus* *Southern*: *Accipiter gentilis*, *Accipiter nisus*, *Aegithalos caudatus*, *Aegolius funereus*, *Alauda arvensis*, *Asio otus*, *Buteo buteo*, *Carduelis spinus*, *Certhia familiaris*, *Columba oenas*, *Corvus corone*, *Delichon urbicum*, *Dryocopus martius*, *Emberiza citrinella*, *Erithacus rubecula*, *Falco tinnunculus*, *Ficedula hypoleuca*, *Fringilla coelebs*, *Glaucidium passerinum*, *Hippolais icterina*, *Loxia curvirostra*, *L. pytyopsittacus*, *Luscinia luscinia*, *Mergus merganser*, *Muscicapa striata*, *Phylloscopus trochilus*, *Regulus regulus*, *Riparia riparia*, *Saxicola rubetra*, *Scolopax rusticola*, *Strix aluco*, *Sylvia communis*, *Turdus viscivorus*, *Turdus philomelos*

^a^Northern species which have declined only in the southern part of their distribution range (Svensson et al. 1999)

### Community change in mammals and functionally associated birds

#### Small herbivores/prey

The small herbivore/prey community is dominated by rodents, in particular bank vole (*Myodes glareolus*), gray-sided vole (*Myodes rufocanus*), field vole (*Microtus agrestis*), and Norwegian lemming (*Lemmus lemmus*). The bank vole is a southern species and field vole is omnipresent, while gray-sided vole and Norwegian lemming are northern species. Their dynamics have been described as generally cyclic with high-amplitude peaks every 3–5 years (Hansson and Henttonen 1985; Angerbjörn et al. 2001). In the twentieth century, however, high-amplitude lemming peaks failed to appear in northern Sweden in 1941–1960 and 1982–2001 (Fig. 2; Angerbjörn et al. 2001). Although an absence of conspicuous high-amplitude peaks does not exclude the possibility that cycles persist, but with inconspicuous low-amplitude peaks, analyses of the dynamics of rodent-dependent predators in 1960–2008 suggest that regular cycles were absent for some time in the 1980s and 1990s in both alpine and boreal rodent communities (Elmhagen et al. 2011). Small mammal trapping data show that cycle amplitude and period declined in boreal voles in 1971–2002, while a pattern with seasonal fluctuations with low spring densities and high autumn densities strengthened (Hörnfeldt 2004). High-amplitude cycles reappeared in 2001, with the exception for gray-sided voles in boreal forests that remained at low population density (Ecke et al. 2010; Elmhagen et al. 2011).

Since the 1970s, cycle amplitude and spring densities in particular have declined in the majority of the studied populations in Europe. The proximate mechanisms causing cycles to fade may vary within Europe as bioclimatic conditions differ. However, climate change is likely to be involved, as cycle amplitude is connected to winter conditions (Cornulier et al. 2013). Substantial snow cover and specific snow characteristics appear to be a prerequisite for high-amplitude peaks in Fennoscandia (Hansson and Henttonen 1985; Kausrud et al. 2008). High-quality snow provides a subnivean space where small rodents can feed and be relatively protected from predators, allowing winter reproduction in lemmings, release from predator control, and rapid population growth (Kausrud et al. 2008; Ims et al. 2011). In the Swedish boreal forest, however, the gray-sided vole also suffers from fragmentation of mature forest (Ecke et al. 2010).

#### Medium-sized herbivores/prey

The medium-sized herbivore/prey community is dominated by mountain hare (*Lepus timidus*) and grouse (capercaillie *Tetrao urogallus*, black grouse *Lyrurus tetrix*, hazel grouse *Tetrastes bonasia*, willow ptarmigan *Lagopus lagopus*, and rock ptarmigan *Lagopus muta*). Although grouse also feed on insects, they are primarily herbivorous. Northern grouse and hare populations generally fluctuate or cycle (Angelstam et al. 1985). In the late nineteenth and early twentieth century, the period of these fluctuations appeared to largely reflect the small rodent cycle, although mountain hare also showed a tendency for longer periods of approximately 10 years (Fig. 2). However, peak abundances seemed to decline in the 1900s in grouse, and after 1930 in hare, possibly with some recovery after 1960. Alternatively, the dynamics may have stabilized temporarily in 1930–1960 (Fig. 2; Sweden’s Official Statistics 1870–1966). Similarly, other sources suggest that rock ptarmigan has declined since the nineteenth century, while capercaillie and black grouse began declining in the 1930s to recover somewhat in the 1960s (Svensson et al. 1999). Both ptarmigan species declined in alpine habitat in the last decade (Lehikoinen et al. 2014). The medium-sized herbivore/prey community also includes some migratory geese. The lesser white-fronted goose (*Anser erythropus*) began declining in alpine Sweden in the 1930s, locally to extinction (Svensson et al. 1999). In at least one site, extirpation occurred within a few years in the 1940s (Ryd 2007).

**Fig. 3:**
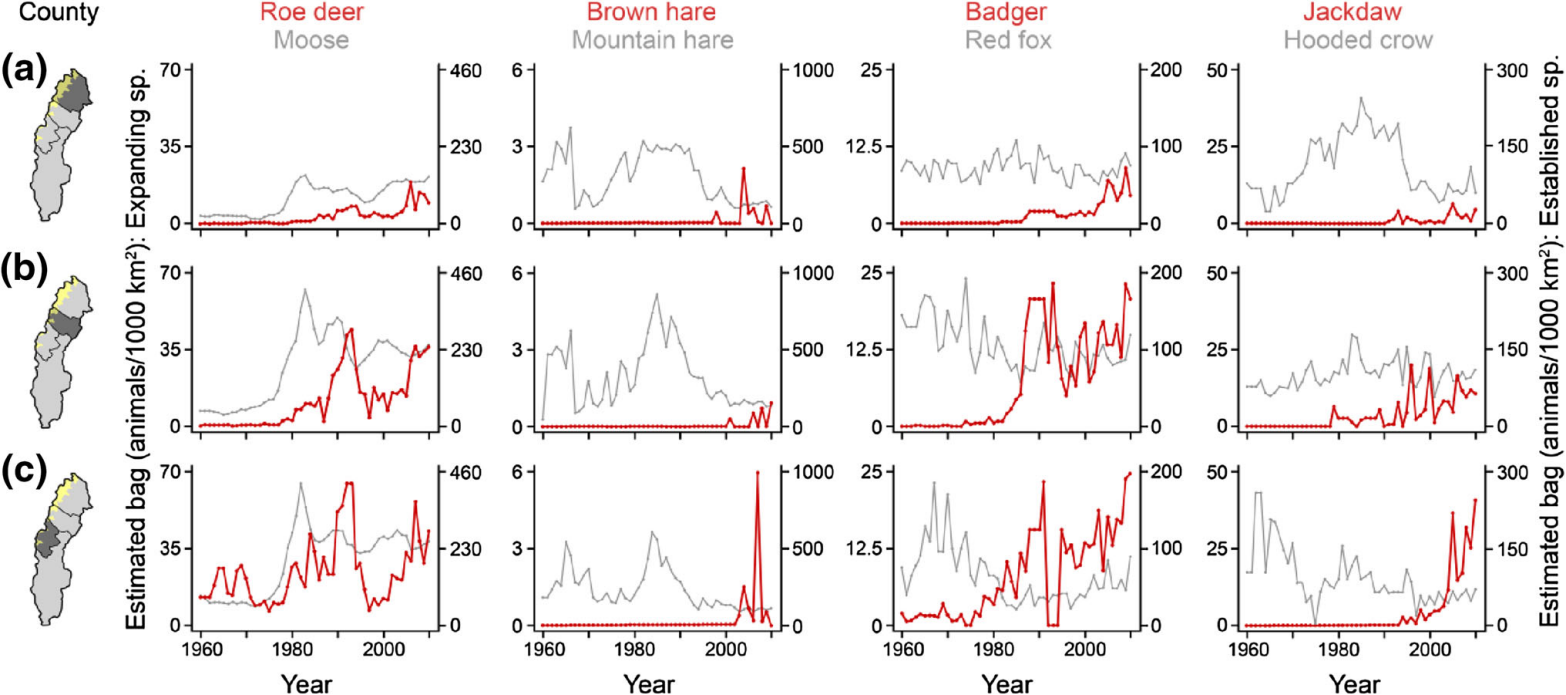
Northern expansion of four southern species (*red*) in the northernmost Swedish counties (**a**–**c**), indicated by hunting bags for 1960–2010, compared to similar northern or already established species (*gray*). The western jackdaw (*Corvus monedula*) was one of the expanding southern bird species (Table 1). Hunting bag data were provided by the Swedish Association for Hunting and Wildlife Management

Early reports attribute low abundance of hare and grouse to unfavorable weather and disease, but increased fox predation is mentioned twice in the 1920s (Sweden’s Official Statistics 1870–1966). At present, the dominant hypothesis suggests that 3–5-year cycles in medium-size herbivores are caused by medium-sized predators, which switch from rodent prey to medium-sized prey in the decrease phase of the rodent cycle (Angelstam et al. 1985; Kausrud et al. 2008). The tendency for 10-year fluctuations in mountain hare might be caused by interactions with larger predators, similar to present-day mountain hare populations in Russian Karelia which fluctuate with an approximate 11-year period tracked by the Eurasian lynx (*Lynx lynx*; Danilov 2010). In Sweden, strong suppression of medium-sized herbivores by red fox predation is supported by a temporary increase in hare and boreal grouse in the 1980s when a mange epizootic reduced the fox population (Fig. 3; Danell and Hörnfeldt 1987; Lindström et al. 1994). Red fox expansion in alpine Sweden is one potential cause of decline in white-fronted goose (Svensson et al. 1999). In Finland, boreal grouse is negatively affected by forestry, climate change, and fox predation (Ludwig 2007). In southern Sweden, the mountain hare has declined due to interference with introduced brown hares (*Lepus europaeus*). Since 1980, the brown hare has advanced north into boreal Sweden, likely in response to mild winters (Fig. 3; Jansson and Pehrson 2007).

#### Large herbivores/prey

**Fig. 4:**
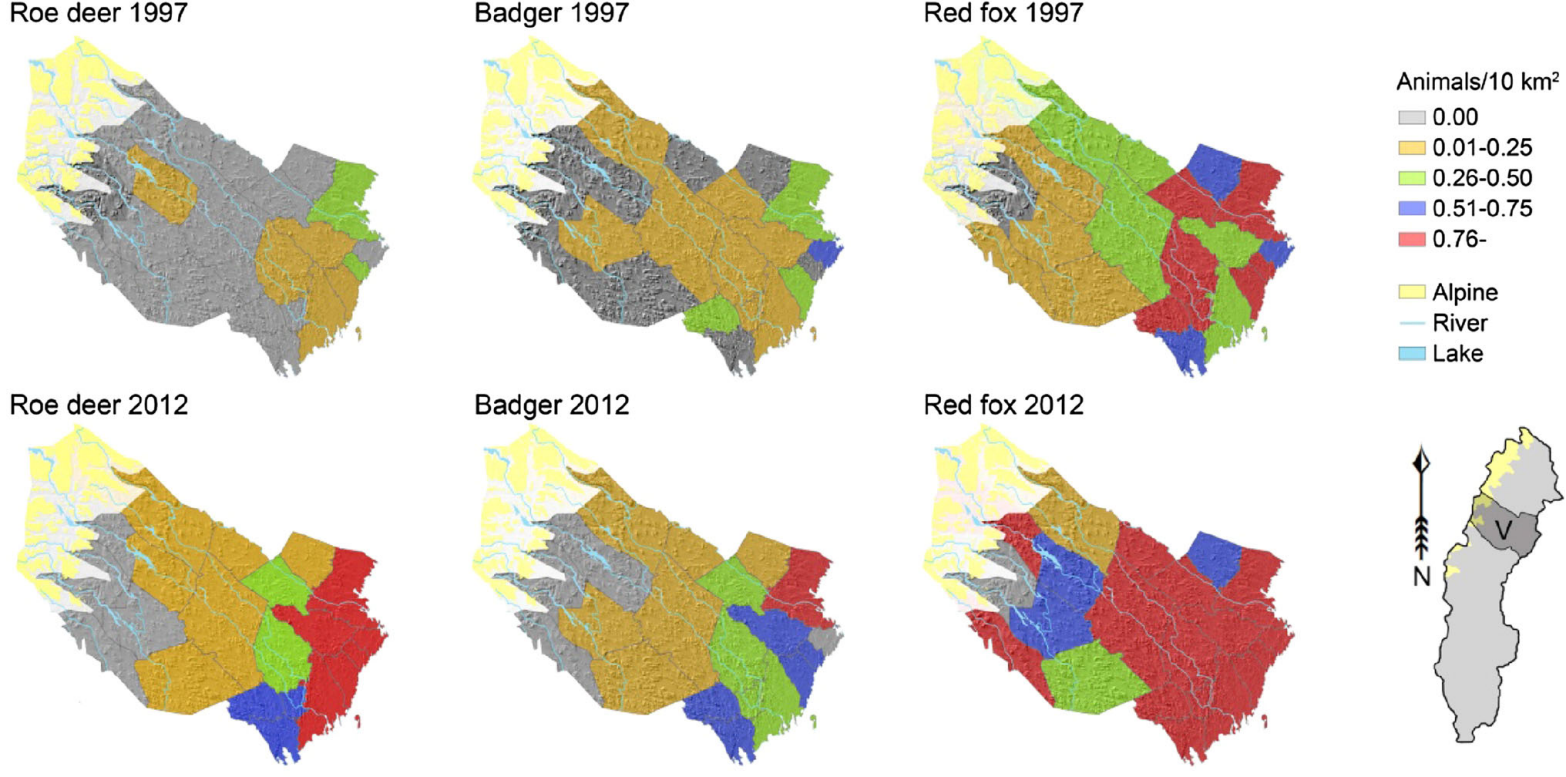
Western expansion or population increase in three southern species between 1997 and 2012, indicated by hunting bags in boreal hunting districts in Västerbotten county (V). The climate is harsher in the west (Fig. 1). Hunting bag data were provided by the Swedish Association for Hunting and Wildlife Management

The guild consists of reindeer, moose (*Alces alces*), and roe deer (*Capreolus capreolus*). All reindeer are semi-domesticated, and the population has fluctuated around 225 000 in the 1900s (Anonymous 2006). Moose declined due to hunting in the early 1800s. By 1830, only a small population remained in south-central Sweden (Liberg et al. 2010). Following protection, moose gradually colonized (recolonized) northern Sweden in 1870–1910 and is now found in all but high alpine areas (Sweden’s Official Statistics 1870–1910; Liberg et al. 2010). In the eighteenth century, the northern distribution limit of roe deer was found in southern boreal Sweden, but the species declined due to hunting in the early 1800s and was extirpated in all but southernmost Sweden (Liberg et al. 2010). Roe deer recolonized temperate Sweden in the 1800s, but then continued to spread north in boreal Sweden in the 1900s. It is now found throughout Sweden, with the exception for alpine tundra and inland boreal forest north of the Arctic Circle (Figs. 3, 4; Sweden’s Official Statistics 1870–1966; Liberg et al. 2010).

Moose were favored by hunting legislation, including different forms of hunting restrictions, large carnivore extirpation, and clear-cutting practices within forestry. These factors also favored roe deer, alongside winter-feeding, milder winters, and the temporary decrease in fox predation during the epizootic in the 1980s (Fig. 3; Liberg et al. 2010).

#### Small and medium-sized predators

There are three southern mammalian predators: badger (*Meles meles*), red fox, and pine marten (*Martes martes*). They have all increased or advanced since the nineteenth century. Pine marten was probably absent for some time between the seventeenth and nineteenth century, but increased in the late nineteenth century and in 1930–1960 (Helldin 2000). The badger had its northern distribution limit in southern boreal Sweden in 1850–1900, but advanced north to the Arctic Circle during the 1900s (Figs. 3, 4; Sweden’s Official Statistics 1870–1966; Bevanger and Lindström 1995). The red fox was repeatedly described as increasing in northern Sweden in 1892–1895, 1914–1920, and 1937–1945. In 1945, it was specifically pointed out that it was spreading to new areas (Sweden’s Official Statistics 1870–1966). The red fox also increased in Norway in 1891–1920, with further increases in 1930–1950 (Selås and Vik 2007).

**Fig. 5:**
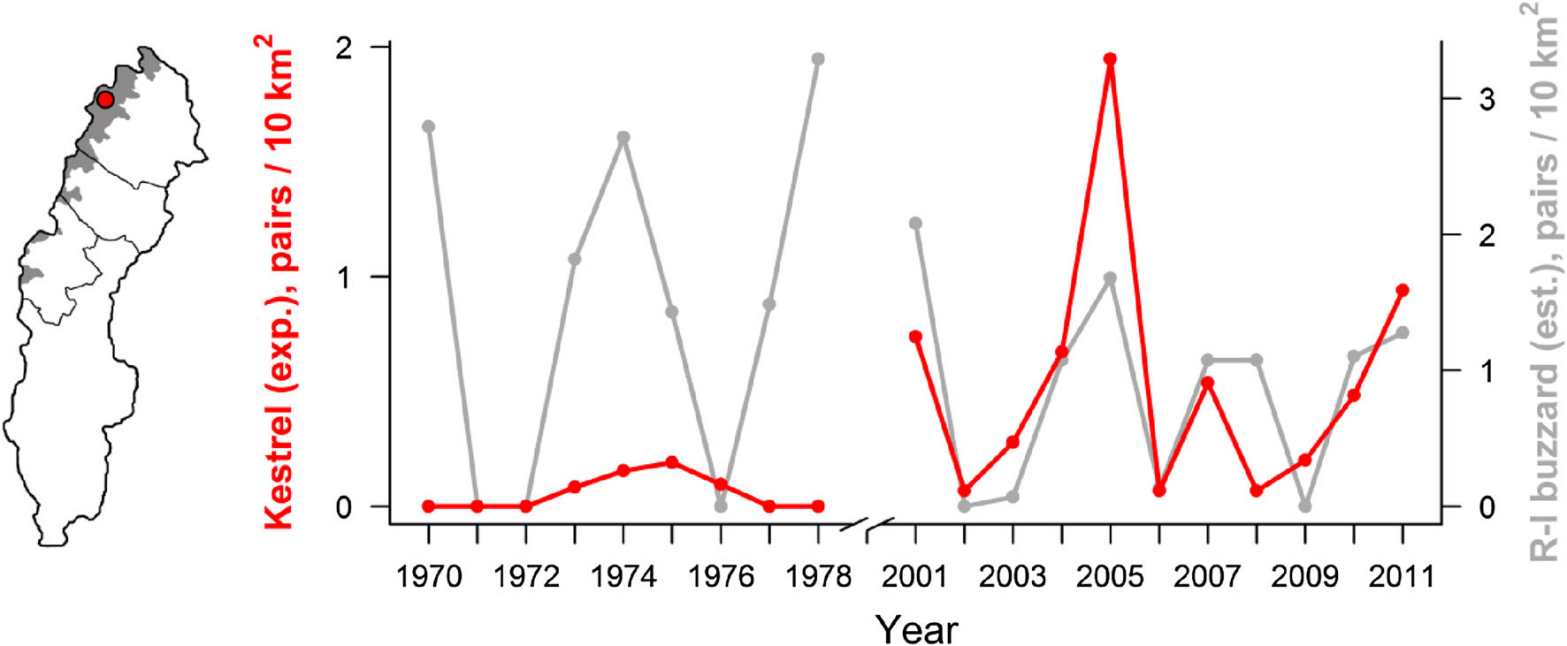
Density of breeding pairs of common kestrel and rough-legged buzzard in Stora Sjöfallet National Park 1970–1978 and 2001–2011. Density estimates include breeders only, i.e., nests where egg clutches were initiated. Both species are rodent specialists at this site, and short-term variation in breeding density is largely explained by numerical responses to small mammal fluctuations. Thus, the density of breeding pairs during rodent peaks is the most reliable population estimate. The rough-legged buzzard declined in Sweden in 1980–2000 (Kjellén and Roos 2000). In Stora Sjöfallet National Park, the average rough-legged buzzard breeding density in rodent peak years was lower in 2001–2011 compared to that in 1970–1978 (this figure)

A large number of birds of prey, as well as the omnivorous gulls, are predators on small rodents (Krebs 2011). The common gull (*Larus canus*) and black-headed gull (*Chroicocephalus ridibundus*) expanded in inland northern Sweden in 1930–1950. The common gull also established in the alpine tundra in the 1960s (Svensson et al. 1999). The Eurasian kestrel (*Falco tinnunculus*) was a rare breeder in at least some mountain regions during the 1970s, but is now at least locally abundant in birch forest and alpine tundra (Fig. 5). In boreal and alpine Sweden, rodent specialists such as northern harrier (*Circus cyaneus*) and rough-legged buzzard (*Buteo lagopus*) declined in 1940–1960 and 1980–2000 (Kjellén and Roos 2000). Likewise, boreal owl (*Aegolius funereus*), short-eared owl (*Asio flammeus*), and long-eared owl (*A. otus*) declined in 1980–2000 (Svensson et al. 1999; Hörnfeldt et al. 2005). The snowy owl (*Bubo scandiaca*) breeds only in alpine tundra. It was probably more common in the southern alpine tundra in the nineteenth century and did not breed at all in Sweden in 1982–2001 (Svensson et al. 1999; Ottvall et al. 2009). The only northern mammalian predator, the arctic fox, was abundant in the alpine tundra until the population plummeted in the early 1900s. Excessive hunting was suggested to cause the decline (Lönnberg 1927), but the arctic fox did not recover despite protection in 1928. The population declined further in 1982–2001 (Angerbjörn et al. 1995, 2013).

There are several suggested drivers of change in the southern species. Pine marten appears sensitive to harvest and the increase in 1930–1960 has been attributed to protection, while the species may have been suppressed by forestry and red fox interactions in the late twentieth century (Helldin 2000). Badger expansion has been suggested to be associated with climate warming, land use promoting young forest stages, reduced hunting pressure, and wolf (*Canis lupus*) extirpation (Bevanger and Lindström 1995). The red fox is favored by climate warming, young forest stages, agricultural land, and anthropogenic infrastructure which provides food sources such as garbage and road kills (Hersteinsson and Macdonald 1992; Kurki et al. 1998; Selås and Vik 2006; Selås et al. 2010). In alpine tundra, the red fox is locally favored by changed reindeer herding practices, where reindeer are kept in the tundra throughout winter which increases the abundance of reindeer carcasses (Killengreen et al. 2011). In southern Sweden, red fox increased in the nineteenth century following large carnivore extirpation, but this effect appeared weak in northern Sweden, potentially due to strong effects of resource limitation (Elmhagen and Rushton 2007).

Declines in specialist rodent predators in northern Sweden in 1982–2001 are linked to poor reproduction due to disrupted rodent dynamics (Angerbjörn et al. 1995; Svensson et al. 1999; Kjellén and Roos 2000; Hörnfeldt et al. 2005; Ottvall et al. 2009). In addition, increased competition with the red fox likely contributed to the decline of the arctic fox in the early 1900s and prevented its recovery (Hersteinsson and Macdonald 1992). The arctic fox has retreated to high-altitude habitats where red fox interactions are least likely to occur (Herfindal et al. 2010). Since 2000, the arctic fox has increased in two Swedish areas with intense conservation efforts, red fox culling, and supplemental feeding, while the number of arctic foxes has remained low in Scandinavian areas without intense actions (Angerbjörn et al. 2013). The positive effect of increased food availability relies on a relative absence of red foxes, as red foxes can monopolize productive habitats and food resources such as carcasses (Killengreen et al. 2007, 2011).

#### Large predators

All large mammalian predators, brown bear (*Ursus arctos*), wolf, Eurasian lynx, and wolverine (*Gulo gulo*), declined due to persecution in the nineteenth and early twentieth century (Sweden’s Official Statistics 1870–1966). The brown bear became restricted to small parts of boreal Sweden, but recovered and spread throughout northern Sweden in the twentieth century (Kindberg et al. 2011). Likewise, wolf became restricted to alpine tundra, where a few individuals remained until extirpation in the 1960s. Wolves recolonized southern Sweden in the 1980s (Wabakken et al. 2001), but the present distribution is restricted by policy to areas south of the reindeer herding area. The southern distribution limit of wolverine, the only northern species, retreated from the southern boreal zone to the alpine zone, where the majority of the population still is found (Flagstad et al. 2004).

Among the large predators, lynx is the only southern species. In the nineteenth century, its northern distribution limit was located slightly south of the Arctic Circle (Sweden’s Official Statistics 1870–1900). This limit was assumed to be determined by climate conditions rather than human influences (Lönnberg 1930). Lynx established and increased in northernmost Sweden in the 1910s, but continued to decline in other parts of Sweden until protection in 1928 (Sweden’s Official Statistics 1870–1966). It has been suggested that lynx was favored by the northern expansion of agriculture and roe deer, as well as large and relatively free-ranging herds of sheep and reindeer, allowing lynx to switch prey from medium-sized herbivores to small ungulates (Sunde et al. 2000). In Russian Karelia and Kola (Fig. 1), the northern distribution limit of lynx is located in the boreal forest slightly north of the Arctic Circle. The more northern distribution in western Fennoscandia has been associated with higher abundance of hare and semi-domesticated reindeer (Danilov 2010). Hare abundance is relatively low in Karelia due to a larger proportion of old-growth forest (Lindén et al. 2000).

## Discussion

In the twentieth century, the northern hemisphere experienced high rates of climate warming primarily before 1940 and after 1980, but there were regional deviations from this trend (IPCC 2013). In northern Sweden, 1930–1960 was the first particularly warm period. This review suggests that the faunal community changed substantially at this time. Climate warming should primarily favor southern species. For the twentieth century as a whole, this was supported by changes in the bird community. Likewise, southern mammals increased and expanded (red fox, pine marten, badger, lynx, moose, and roe deer), while northern mammals retreated and declined (gray-sided vole, arctic fox, and wolverine). These observations are in line with the hypothesis that climate warming alters northern ecosystems. However, several of these changes have been suggested to be caused by drivers such as land-use change, anthropogenic food subsidies, and hunting. It is possible that the potential effects of climate warming were overlooked until recently, when the research focus shifted toward this driver. For example, recent findings show that the abundance composition of the Swedish bird community has tracked the temperature trend since the 1960s (Lindström et al. 2013), and several birds have changed their phenology in the last century, although this pattern appears stronger in southern than in northern Sweden (Kullberg et al. 2015). Furthermore, out of 14 investigated alpine bird species, 9 declined significantly in 2002–2012, concurrent with increasing summer temperature and precipitation (Lehikoinen et al. 2014). Likewise, one-third of the alpine specialists, i.e., species that primarily breed in alpine areas, declined over the last 30 years (Ottvall et al. 2009). Nevertheless, we suggest that the information in this review, taken together, indicates that climate warming worked in synergy with other factors, and that all these drivers primarily favored southern species.

Although there is substantial local variation, the altitudinal treeline in Sweden has advanced by an average 70–90 m in the twentieth century, and forest densification and increased shrub cover have been observed in some forest-to-tundra borderlands (Kullman and Öberg 2009; Callaghan et al. 2013). In the Arctic tundra, large herbivores in particular may escape predator regulation. However, with increasing summer temperature, the strength of herbivory decreases while that of predation increases, suggesting that the climate warming may cause a shift from bottom-up to top-down regulation of herbivores (Legagneux et al. 2014). In Sweden, reindeer grazing has remained strong in alpine habitat, favoring graminoids, suppressing dwarf shrubs, and at least locally buffering against climate-driven treeline rise (Olofsson et al. 2004; Van Bogaert et al. 2011). In Canada, most caribou (i.e., reindeer) populations are declining at present. Unfavorable weather conditions have had a negative effect on one caribou population, but land-use changes which indirectly favor large carnivores and increase the predation pressure on caribou are the main driver of these declines (Festa-Bianchet et al. 2011). In Sweden, feeding of semi-domesticated reindeer during adverse winters and management of large predators may have prevented such change. Small rodent grazing can also have strong effects on the alpine plant community (Olofsson et al. 2012), but grazing effects should have weakened during periods without high-amplitude population peaks.

The small rodent cycle, and associated cycles in rodent predators and alternative prey, is a key characteristic of northern ecosystems (Ims and Fuglei 2005; Krebs 2011). However, temporary release from top-down control is necessary for rodents to reach high-amplitude peaks (Ims et al. 2011; Legagneux et al. 2012). Hence, although fading cycles have been related to direct effects of snow characteristics (Kausrud et al. 2008), climate change should also entail northern advancement of generalist predators that should stabilize the rodent cycle (Hanski et al. 1991). Fading rodent cycles could also have positive feedback effects on generalist predators. Non-cyclic dynamics provide a more stable food resource, allowing some reproduction in all years (Hörnfeldt et al. 2005). Specialist rodent predators, which rely on high-amplitude peaks for breeding, could suffer from this development or be outcompeted by generalists (Ims and Fuglei 2005; Henden et al. 2009). Similarly, the recent expansion of kestrels in some boreal–alpine borderlands may be facilitated by declines in larger competitors in the rodent specialist guild, e.g., the rough-legged buzzard (Fig. 5). However, this trend can also partly be explained by an increase in foraging habitat related to clear-cutting practices, and higher availability of nest sites caused by large-scale nest box programs (e.g., Saurola 2012).

We suggest that climate warming has worked synergistically with other long-term anthropogenic drivers. For example, historical land use might have allowed some northern birds to expand into marginal southern habitats (Svensson et al. 1999), but they could be highly sensitive to climate change in these localities. Likewise, resource subsidies, such as alternative habitat or food, can increase and stabilize the dynamics of southern species in marginal northern habitat, providing a buffer against adverse periods (Henden et al. 2009). In Fennoscandia, Karelia retains a relatively large proportion of old-growth boreal forest (Lindén et al. 2000). Roe deer was absent in the relatively cold period 1700–1900, but recolonized along rivers in the early 1900s. However, it remains restricted to watersides, agricultural land, and clear-cuts (Danilov 2010). Expansion of agriculture, forestry, built-up areas, and other infrastructure in Sweden likely provided southern species with dispersal corridors and suitable habitat, as well as refugia during adverse times. Thus, the northern advance in southern species that we observed in the 1900s could be seen as classic habitat tracking (Darwin 1859). Land-use change increased the structural and functional connectivity for southern generalists, as well as the temporal connectivity by providing refugia during less-favorable climate periods (Auffret et al. 2015). This should have facilitated and reinforced northern expansions.

While synergistic negative effects of multiple drivers of change are a common problem for threatened and declining species (Brook et al. 2008), synergistic positive effects of climate and land-use change can favor invasive species. It has also been suggested that positive synergies may facilitate the expansion of temperate species to higher altitudes (Bellard et al. 2013). Outside Fennoscandia, land-use change has so far had relatively little impact on the northern boreal and tundra biome, as these remain relatively unexploited in large parts of Russia and Canada (Brooks et al. 2006). However, our study highlights that positive and negative synergies between climate warming and land-use change may be wide-spread in northern biomes experiencing both forms of change. Climate and land-use change are both projected to become strong drivers of biodiversity change in the boreal biome over the next century (Sala et al. 2000), suggesting that the impact of these synergies will increase substantially in the northern hemisphere.

## Conclusion

We suggest that the alpine and boreal ecosystems in Sweden have responded to climate warming, and that substantial changes occurred already in 1930–1960. Observed changes include range expansions and increases in southern species, retreat and decline in northern species, as well as changing dynamics. However, land-use change and other anthropogenic drivers probably worked in synergy with climate warming. Human use of boreal and Arctic ecosystems is likely to intensify with climate warming. Thus, understanding change in northern ecosystems requires a broad research approach that includes human use of the ecosystem. Future research should aim to separate effects of climate warming, land-use change, and other anthropogenic drivers.

## Electronic supplementary material

Below is the link to the electronic supplementary material.

Supplementary material 1 (PDF 135 kb)
