# Supplementary material for: A boreal invasion in response to climate change? Range shifts and community effects in the borderland between forest and tundra
Source: Ambio. 2015 Jan 9;44(Suppl 1):39–50. doi: 10.1007/s13280-014-0606-8 (PMC4289007; doi:10.1007/s13280-014-0606-8)
Supplement: Supplementary file 1 — Supplementary material 1 (PDF 135 kb) [file 13280_2014_606_MOESM1_ESM.pdf]

AMBIO

Electronic supplementary material

This supplementary material has not been peer reviewed.

A boreal invasion in response to climate change? Range shifts and community effects in the borderland between forest and tundra

Bodil Elmhagen, Jonas Kindberg, Peter Hellström, Anders Angerbjörn

MATERIALS AND METHODS – EXTENDED VERSION

In this paper, we review observed changes in the distribution, abundance and dynamics of birds and mammals in boreal and alpine Sweden since the 19th century, as well as suggested drivers of change.

The first part of the review is an overview of known changes in southern and northern birds. According to our definition (see paper), 118 of the 196 species which regularly breed in boreal or alpine Sweden had a southern or northern distribution (Table S1). The remaining 78 species, i.e. the ones not included in the paper (Table S2), were species which did not have one distinct latitudinal or altitudinal distribution limit in boreal or alpine Fennoscandia. Their distribution could instead be, for example, eastern or western (i.e. they have both a southern and a northern distribution limit in boreal/alpine Fennoscandia), patchy (e.g. including both alpine tundra and coasts), coastal, omnipresent (i.e. the distribution covered all of Fennoscandia), or a distribution caused by an introduction or large-scale European range expansion during the 20th century which meant that the species was not present at the beginning of the study period (Table S2). To not exclude southern species which might have expanded north throughout Sweden since the 19th century and at present consequently have a northern distribution limit slightly north of Sweden, we classified species as southern based on their northern distribution limit in Fennoscandia. However, our source of information on long-term range shifts and population trends in Swedish birds (Svensson et al. 1999), only reported the present distribution within Sweden. We therefore based our classification on distribution maps in Grant and Svensson (1999). For Sweden, the distribution maps in Svensson et al. (1999) correspond highly with those in Grant and Svensson (1999).

Svensson et al. (1999) compiled quantitative and qualitative information on

Swedish bird species, including a section on the history of each species since the 19th century. For each of the 118 southern and northern species, we used the historical information provided by Svensson et al. (1999) to assess long-term trends, defined as the net change in the range or abundance of each species between the 19th century and late 20th century. Firstly, we noted whether the distribution limit was known to have changed (range contraction or expansion). Such information on range shifts were found for 6 northern and 28 southern species. Secondly, if the distribution limit had not changed, we noted whether the information provided could be interpreted as a net change in abundance (long-term increasing or declining trend). This was the case for 11 northern and 23 southern birds. For one declining species (*Charadrius morinellus*), this trend classification was based on sources from Finland, which substantiated a general declining trend in Europe. For all other species, the trend classification was based on information from Sweden. We classified the remaining 50 species as having "no long-term trend", which meant that we could not interpret the provided information as an overall increase or decline. This category included (1) species where the population size had been stable; (2) species which had experienced fluctuations without a directional trend, e.g. decline(s) followed by recovery; (3) species where there had been locally different trends within the study area; (4) species where no information was available.

The second part of the review includes information which we assessed from yearly reports on forestry published by Sweden's Official Statistics between 1870 and 1966. We refer to these reports as one source (Sweden's Official Statistics 1870-1966). The reports summarize information provided by e.g. foresters to the person(s) writing the reports. The contents vary somewhat over time, suggesting it was partly up to the writer(s) to decide what information to include. In most years, however, the reports include brief qualitative statements on the status of game species and predators

in different parts of Sweden. For example, it could be stated in which parts of Sweden a persecuted species still was found, or which areas an expanding species had reached. For game species, the availability in a particular district, county or over a larger region could be described as e.g. "plenteous", "abundant", "good", "average", "scanty", "abysmal", "increasing/declining since last year", or "no change since last year". We refer to the reports both to describe change in species distributions, and to describe the local dynamics of some game species in the county of Västerbotten in 1870-1966. For the latter, we reclassified the qualitative descriptions of the status of each species each year into five categories; abundant, good, average, below average and scarce. In years when a species was described as increasing or decreasing since last year, we changed the classification one step up or down, respectively. If a species was described as increasing or decreasing for several years, we extrapolated the yearly status during this time period. For example, if the availability of a species was described as scarce in year 1, increasing in the next three years, and then as average in year 5, we extrapolated the degree of increase from scarce to average in the intermediate years. Regarding the quality of the reports, we think it is reasonable to assume that the overall picture is reliable, although the accuracy of each individual piece of information probably varies depending on the meticulousness of individual reporters and writers.

To illustrate temperature trends in northern Sweden, we retrieved temperature data from the Swedish Meteorological and Hydrological Institute. Four weather stations were operating continuously from 1862 to the early 2000s. We used this data to calculate a 10-year running mean in yearly temperature.

We use data from a survey of a raptor guild in Stora Sjöfallet National Park (67°29′N 18°21′E) to illustrate potential northern advancement in Eurasian kestrels (*Falco tinnunculus*) since the 1970s. For comparison, we also showed data on rough-

legged buzzards (*Buteo lagopus*). Surveys were carried out at this location, comprising the same nest sites, in 1970-1978 and 2001-2011 (Broo and Lindberg 1981, Hellström 2014). The densities of the two species were estimated as the number of breeding pairs per 10 km^2, where breeding pairs were defined as pairs which initiated egg clutches. Number of breeding pairs varied between 0 to 35 for kestrels and 0 to 70 for rough legged buzzards.

We exemplify northern and northwestern advancement in game species using estimated hunting bags provided by the Swedish Association for Hunting and Wildlife Management. The association collects yearly reports from hunters throughout Sweden and estimate regional hunting bags by extrapolating the number of animals killed in reported areas to the area of counties (Elmhagen et al. 2011). Since 1997, hunting bags are also estimated for hunting districts within counties.

References

Broo, B., and P. Lindberg. 1981. Exploitation of the Ritsem area – effects on the fauna, especially raptors. Statens naturvårdsverk, Solna. (in Swedish)

Dyntaxa (2013) Swedish Taxonomic Database. Accessed at http://www.dyntaxa.se/taxon/ at 02-12-2014.

Hellström, P. 2014. Predator responses to non-stationary rodent cycles. PhD thesis, Stockholm University.

Grant, P.J., and L. Svensson (ed.). 1999. *The Bird Guide. Europe and Mediterranean birds in the field*. Albert Bonniers Förlag, Stockholm, Sweden. (in Swedish)

Svensson, S., M. Svensson, and M. Tjernberg. 1999. *Swedish Bird Atlas*. The Swedish Species Information Centre and the Sweden's Ornithological Society. (In Swedish)

Sweden's Official Statistics. 1870-1966. Forestry. Yearly reports. Norstedt & Söner,

Stockholm, Sweden. (in Swedish)

Table S1. The 118 species that were classified as having either a southern (S) or northern (N) distribution in boreal/alpine Sweden. For each species, the change assessed from Svensson et al. (1999) is described as a range shift (contraction or expansion), a long-term trend in abundance (increase or decline) or as no change (none). The category "no change" includes both species which did not show a long-term change in abundance (see text) and species for which no data were available. For all species which showed either a range shift or a long-term trend, suggested drivers of change were assessed from Svensson et al. (1999). Nomenclature following Dyntaxa (2013).

**Species**	**Common name**	**S or N**	**Change**	**Suggested drivers of change**
Accipiter gentilis	northern goshawk	S	None	
Accipiter nisus	Eurasian sparrowhawk	S	None	
Acrocephalus scirpaceus	Eurasian reed warbler	S	Expansion	land-use change (agriculture and eutrophication)
Aegithalos caudatus	long-tailed bushtit	S	None	
Aegolius funereus	boreal owl	S	None	
Alauda arvensis	Eurasian skylark	S	None	
Anas crecca	Eurasian teal	S	Decline	land-use change (drainage of wet habitats)
Anas penelope	Eurasian wigeon	N	None	
Anas platyrhynchos	mallard	S	Increase	unknown
Anser erythropus	lesser white-fronted goose	N	Decline	hunting, predation, change in overwintering areas
Anser fabalis	bean goose	N	Decline	hunting, land-use change (agriculture and drainage of wet habitats)
Anthus cervinus	red-throated pipit	N	None	
Anthus trivialis	tree pipit	S	Decline	land-use change (agriculture), change in overwintering areas
Apus apus	common swift	S	Increase	other human-related factors (more nesting sites)
Asio otus	long-eared owl	S	None	
Aythya marila	greater scaup	N	Decline	unknown
Bubo scandiacus	snowy owl	N	Contraction	disrupted rodent dynamics
Buteo buteo	common buzzard	S	None	
Buteo lagopus	roughleg	N	None	
Calcarius lapponicus	Lapland longspur	N	None	
Calidris alpina alpina	dunlin	N	None	
Calidris falcinellus	broad-billed sandpiper	N	Contraction	land-use change (agriculture) and drainage of wet habitats
Calidris maritima	purple sandpiper	N	None	
Calidris temminckii	Temminck's stint	N	Decline	land-use change (agriculture)
Caprimulgus europaeus	European nightjar	S	Decline	land-use change (agriculture), change in overwintering areas
Carduelis cannabina	common linnet	S	Decline	land-use change (agriculture)
Carduelis chloris	European greenfinch	S	Expansion	other human-related factors (e.g. favored by feeding and associated with built-up areas)
Carduelis flammea flammea	common redpoll	N	None	
Carduelis hornemanni	Arctic redpoll	N	None	
Carduelis spinus	Eurasian siskin	S	None	
Certhia familiaris	Eurasian treecreeper	S	None	
Charadrius dubius	little ringed plover	S	Expansion	other human-related factors (associated with exploited areas)
Charadrius morinellus	Eurasian dotterel	N	Decline	hunting, change in overwintering areas
Chroicocephalus ridibundus	black-headed gull	S	Expansion	unknown
Clangula hyemalis	long-tailed duck	N	None	
Columba oenas	stock dove	S	None	

Columba palumbus	common wood pigeon	S	Increase	land-use change (agriculture), other human-related factors (associated with built-up areas)
Corvus corone	hooded crow	S	None	
Corvus monedula	western jackdaw	S	Expansion	land-use change (agriculture), other human-related factors (associated with built-up areas)
Cuculus canorus	common cuckoo	S	Decline	unknown
Cyanistes caeruleus	Eurasian blue tit	S	Expansion	land-use change (agriculture), other human-related factors (e.g. favored by built-up areas, feeding and nesting boxes)
Cygnus olor	mute swan	S	Expansion	land-use change causing eutrophication, climate change
Delichon urbicum	common house martin	S	None	
Dendrocopos major	great spotted woodpecker	S	Increase	land-use change (forestry), other human-related factors (feeding)
Dendrocopos minor	lesser spotted woodpecker	S	Decline	land-use change (forestry)
Dryocopus martius	black woodpecker	S	None	
Emberiza citrinella	yellowhammer	S	None	
Eremophila alpestris	horned lark	N	Decline	unknown
Erithacus rubecula	European robin	S	None	
Falco columbarius	merlin	N	Decline	land-use change (agriculture)
Falco rusticolus	gyrfalcon	N	None	
Falco tinnunculus	common kestrel	S	None	
Ficedula hypoleuca	European pied flycatcher	S	None	
Fringilla coelebs	chaffinch	S	None	
Fulica atra	Eurasian coot	S	Expansion	land-use change (drainage of wet habitats specifically lowered water level in lakes, eutrophication)
Gallinago media	great snipe	N	Contraction	land-use change (drainage of wet habitats), hunting
Garrulus glandarius	Eurasian jay	S	Expansion	unknown
Gavia stellata	red-throated loon	N	Decline	land-use change (agriculture, drainage of wet habitats)
Glaucidium passerinum	Eurasian pygmy owl	S	None	
Hippolais icterina	icterine warbler	S	None	
Hirundo rustica	barn swallow	S	Decline	land-use change (agriculture)
Jynx torquilla	Eurasian wryneck	S	Decline	land-use change (agriculture)
Lagopus lagopus	willow ptarmigan	N	Contraction	unknown
Lagopus muta	rock ptarmigan	N	Decline	reindeer grazing
Lanius collurio	red-backed shrike	S	Decline	land-use change (agriculture), change in overwintering areas
Larus canus	mew gull	S	Expansion	unknown
Limosa lapponica	bar-tailed godwit	N	None	
Lophophanes cristatus	European crested tit	S	Expansion	climate change
Loxia curvirostra	red crossbill	S	None	
Loxia pytyopsittacus	parrot crossbill	S	None	
Luscinia luscinia	thrush nightingale	S	None	
Luscinia svecica	bluethroat	N	None	
Lyrurus tetrix	black grouse	S	Decline	land-use change (drainage of wet habitats)
Melanitta nigra	black scoter	N	None	
Mergus merganser	common merganser	S	None	
Mergus serrator	red-breasted merganser	S	Decline	land-use change causing eutrophication
Motacilla flava thunbergi	yellow wagtail	S	Increase	land-use change (forestry)
Muscicapa striata	spotted flycatcher	S	None	
Numenius arquata	Eurasian curlew	S	Expansion	land-use change (agriculture, drainage of wet habitats)

Numenius phaeopus	whimbrel	N	Expansion	unknown
Parus major	great tit	S	Expansion	unknown
Passer domesticus	house sparrow	S	Decline	land-use change (agriculture)
Passer montanus	Eurasian tree sparrow	S	Expansion	other human-related factors (associated with gardens)
Periparus ater	coal tit	S	Expansion	land-use change (forestry)
Pernis apivorus	European honey buzzard	S	Decline	change in overwintering areas
Phalaropus lobatus	red-necked phalarope	N	None	
Phoenicurus phoenicurus	common redstart	S	Decline	Land-use change (forestry)
Phylloscopus sibilatrix	wood warbler	S	Expansion	unknown
Phylloscopus trochilus	willow warbler	S	None	
Pica pica	Eurasian magpie	S	Expansion	land-use change (agriculture), other human-related factors (decreased hunting)
Picus viridis	European green woodpecker	S	Decline	land-use change (agriculture, forestry)
Plectrophenax nivalis	snow bunting	N	None	
Pluvialis apricaria	European golden plover	N	Decline	land-use change (agriculture, forestry)
Podiceps cristatus	great crested grebe	S	Expansion	Land-use change (eutrophication)
Poecile montanus	willow tit	S	Decline	land-use change (forestry)
Prunella modularis	dunnock	S	Expansion	land-use change (forestry)
Pyrrhula pyrrhula	Eurasian bullfinch	S	Expansion	unknown
Regulus regulus	goldcrest	S	None	
Riparia riparia	sand martin	S	None	
Saxicola rubetra	whinchat	S	None	
Scolopax rusticola	Eurasian woodcock	S	None	
Sitta europaea	Eurasian nuthatch	S	Expansion	unknown
Stercorarius longicaudus	long-tailed jaeger	N	None	
Strix aluco	tawny owl	S	None	
Sturnus vulgaris	common starling	S	Expansion	land-use change (agriculture)
Sylvia atricapilla	Eurasian blackcap	S	Expansion	land-use change (agriculture)
Sylvia borin	garden warbler	S	Expansion	unknown
Sylvia communis	common whitethroat	S	None	
Sylvia curruca	lesser whitethroat	S	Expansion	land-use change (forestry)
Tetrao urogallus	western capercaillie	S	Decline	land-use change (drainage of wet habitats, forestry), predation
Tringa glareola	wood sandpiper	N	Decline	land-use change (drainage of wet habitats)
Tringa ochropus	green sandpiper	S	Expansion	unknown
Troglodytes troglodytes	Eurasian wren	S	Increase	land-use change (forestry), climate change
Turdus merula	common blackbird	S	Expansion	unknown
Turdus philomelos	song thrush	S	None	
Turdus torquatus	ring ouzel	N	Contraction	land-use change (agriculture)
Turdus viscivorus	mistle thrush	S	None	
Vanellus vanellus	northern lapwing	S	Expansion	unknown

Table S2. The 78 species breeding in alpine or boreal Sweden that did not have a southern or northern distribution according to our definition, and thus were excluded from part one of the review. Nomenclature following Dyntaxa (2013).

**Species**	**Common name**	**Distribution**
Acrocephalus schoenobaenus	sedge warbler	patchy
Actitis hypoleucos	common sandpiper	omnipresent
Alca torda	razorbill	coastal
Anas acuta	northern pintail	patchy
Anas querquedula	garganey	patchy
Anser anser	greylag goose	coastal
Anthus petrosus	rock pipit	coastal
Anthus pratensis	meadow pipit	omnipresent
Aquila chrysaetos	golden eagle	patchy
Arenaria interpres	ruddy turnstone	coastal
Asio flammeus	short-eared owl	eastern
Aythya ferina	common pochard	eastern-coastal
Aythya fuligula	tufted duck	eastern-patchy
Bombycilla garrulus	Bohemian waxwing	eastern
Branta canadensis	Canada goose	introduced during study period
Bubo bubo	Eurasian eagle-owl	eastern-patchy
Bucephala clangula	common goldeneye	eastern
Calidris pugnax	ruff	eastern-patchy
Carpodacus erythrinus	common rosefinch	eastern-patchy
Cepphus grylle	black guillemot	coastal
Charadrius hiaticula	common ringed plover	coastal-patchy
Cinclus cinclus	white-throated dipper	western (both southern and northern distribution limits)
Circus cyaneus	northern harrier	eastern
Corvus corax	northern raven	omnipresent
Corvus frugilegus	rook	patchy
Cygnus cygnus	whooper swan	eastern
Emberiza hortulana	ortolan bunting	eastern
Emberiza pusilla	little bunting	eastern-patchy (colonized during the 20th century)
Emberiza rustica	rustic bunting	eastern
Emberiza schoeniclus	reed bunting	omnipresent
Falco peregrinus	peregrine falcon	eastern-patchy
Falco subbuteo	Eurasian hobby	eastern
Fringilla montifringilla	brambling	eastern
Gallinago gallinago	common snipe	omnipresent
Gavia arctica	black-throated loon	southern distribution limit south of boreal Sweden
Grus grus	common crane	eastern
Haematopus ostralegus	Eurasian oystercatcher	coastal
Haliaeetus albicilla	white-tailed eagle	patchy
Hydrocoloeus minutus	little gull	patchy
Hydroprogne caspia	Caspian tern	coastal
Lanius excubitor	great grey shrike	eastern
Larus argentatus	European herring gull	coastal-patchy
Larus fuscus	lesser black-backed gull	coastal
Larus marinus	great black-backed gull	coastal
Locustella naevia	common grasshopper warbler	eastern (colonized during the 20th century)
Lymnocryptes minimus	jack snipe	eastern
Melanitta fusca	velvet scoter	coastal-patchy
Mergellus albellus	smew	eastern
Motacilla alba alba	white wagtail	omnipresent
Motacilla cinerea	grey wagtail	colonized Sweden in the 20th century
Numenius phaeopus	whimbrel	eastern

Oenanthe oenanthe	northern wheatear	omnipresent
Pandion haliaetus	osprey	eastern
Perisoreus infaustus	Siberian jay	eastern
Phylloscopus borealis	Arctic warbler	eastern
Phylloscopus collybita	common chiffchaff	patchy
Picoides tridactylus	Eurasian three-toed woodpecker	eastern
Picus canus	grey-headed woodpecker	eastern-patchy
Pinicola enucleator	pine grosbeak	eastern
Podiceps auritus	horned grebe	eastern-patchy
Podiceps grisegena	red-necked grebe	patchy
Poecile cinctus	Sibirian tit	eastern
Regulus regulus	goldcrest	eastern-patchy
Somateria mollissima	common eider	coastal
Stercorarius parasiticus	parasitic jaeger	coastal
Sterna hirundo	common tern	coastal-patchy
Sterna paradisaea	Arctic tern	coastal-patchy
Streptopelia decaocto	Eurasian collared dove	colonized Sweden in the 20th century
Strix nebulosa	great grey owl	eastern-patchy
Strix uralensis	Ural owl	eastern
Surnia ulula	northern hawk-owl	eastern
Tetrastes bonasia	hazel grouse	eastern
Tringa erythropus	spotted redshank	eastern
Tringa nebularia	common greenshank	eastern
Tringa totanus	common redshank	patchy
Turdus iliacus	redwing	southern distribution limit south of boreal Sweden
Turdus pilaris	fieldfare	omnipresent-eastern (20th century western range expansion in Europe)
Uria aalge	common murre	coastal
